# Feasibility and Acceptability of AI-Powered Tools for Early Autism Screening in Egypt: Semistructured Focus Group Study

**DOI:** 10.2196/82564

**Published:** 2026-04-07

**Authors:** Pratheepan Yogarajah, Ammal M Metwally, Priyanka Chaurasia, Ghada A Elshaarawy, Shereen M El Khateeb, Engy A Ashaat, Amal Elsaeid, Nahed A Elghareeb, Amira S ElRifay

**Affiliations:** 1 School of Computing, Engineering & Intelligent Systems, Ulster University Londonderry, Northern Ireland United Kingdom; 2 National Research Centre Giza, Giza Egypt; 3 Department of psychology, American University in Cairo Cairo Egypt; 4 Ministry of Health and Population Cairo Egypt

**Keywords:** AI, artificial intelligence, ASD, autism spectrum disorder, developing countries, Egypt, health equity, mobile applications, qualitative research, screening

## Abstract

**Background:**

Autism spectrum disorder (ASD) is often underdiagnosed in low- and middle-income countries due to limited specialist access, sociocultural stigma, and fragmented screening systems. Artificial intelligence (AI)–powered screening tools may improve early detection by enabling low-cost, accessible assessments. However, adoption depends on stakeholder trust, ethical safeguards, and alignment with local health system capacities.

**Objective:**

This study explored the feasibility, acceptability, and perceived ethical and practical enablers and barriers to implementing AI-powered tools for early ASD screening in Egypt, with attention to urban–rural disparities and integration into existing care pathways.

**Methods:**

We used a qualitative design with semistructured focus group discussions with 49 participants (21 parents of children with ASD and 28 health care professionals) recruited from urban and rural governorates. Discussions were audio-recorded, transcribed verbatim, and analyzed using Braun and Clarke’s reflexive thematic analysis, supported by NVivo software (Lumivero). Methodological integrity was ensured through reflexivity, triangulation, and peer debriefing. Thematic saturation was monitored across groups, and participant diversity was prioritized across contexts.

**Results:**

Five themes emerged: (1) AI as a supportive tool rather than a replacement for clinicians, emphasizing scalability and assistance for nonspecialists; (2) the need for cultural and contextual adaptation to ensure local relevance; (3) privacy, trust, and transparency concerns, including data security, consent, and algorithmic opacity; (4) reducing diagnostic inequities by addressing urban–rural disparities and strengthening community-based deployment; and (5) the preference for hybrid AI–human models, with conditions for adoption including cultural sensitivity, human oversight, and digital literacy support. Counts (n/N) of parents and health care professionals contributing to each theme were used descriptively as indicators of pattern salience rather than as statistical estimates of prevalence. Participants expressed cautious optimism, with parents emphasizing accessibility and speed, while health care professionals highlighted concerns about reliability, cultural adaptation, and data governance.

**Conclusions:**

AI-powered ASD screening has potential to advance equitable early detection in underserved areas. Adoption requires transparent data governance, integration into hybrid human–AI models, culturally adaptive design, and targeted digital literacy initiatives. These findings provide an evidence-based roadmap for policymakers, technologists, and health system leaders to implement AI screening tools that are ethically sound, contextually relevant, and equity-focused.

## Introduction

Autism spectrum disorder (ASD) is a prevalent neurodevelopmental condition that affects cognitive, behavioral, and social functioning, necessitating early and accurate identification for optimal developmental outcomes [1,2]. Early detection is particularly important because timely intervention can substantially improve language development, social skills, and long-term adaptive functioning [3]. However, many children remain undiagnosed due to barriers such as limited specialist access, cultural stigma, and inconsistent screening practices, particularly in low-resource settings [4,5].

Traditional screening methods, including clinical observation, structured interviews, and standardized behavioral checklists, remain the cornerstone of ASD diagnosis but are constrained by subjectivity and poor scalability. In Egypt, where the estimated prevalence of children aged 2-12 years at high risk for ASD is 3.3% (95% CI 3.1%-3.5%) [6] and those diagnosed with autism is 1.1% (95% CI 1.0%-1.2%) [7], the burden of undiagnosed developmental conditions is compounded by delayed health-seeking and shortages of trained professionals [6]. Developmental delays affect 6.4% of preschool-aged children [6] and 7.4% of school-aged children, underscoring the urgent need for scalable and accessible diagnostic alternatives [8]. These figures highlight the systemic challenges faced by families and health systems, where demand for developmental assessment far exceeds available capacity.

Artificial intelligence (AI) has emerged as a promising adjunct to traditional screening by enhancing diagnostic accuracy and reducing reliance on clinical judgment [9]. Machine learning models have demonstrated potential in identifying ASD-related traits via speech, behavior, facial analysis, and digital interactions [10]. Mobile AI apps, in particular, offer low-cost, early-stage screening accessible to both parents and frontline health care providers, especially in underserved communities [11,12]. Such tools could bridge gaps in access and standardization, addressing the fragmented diagnostic pathways observed in Egypt and other low- and middle-income countries (LMICs) [13].

Despite their promise, AI-driven tools face challenges, including algorithmic bias, data privacy concerns, limited cultural adaptability, and stakeholder mistrust [14]. In addition, most existing AI models are trained on Western datasets, raising questions about their validity and cultural fit in LMIC contexts [15]. Broader digital health research also shows that successful scale-up in low-resource environments requires attention to cultural adaptation, stakeholder engagement, and health system integration [16]. The feasibility and success of integrating AI into ASD screening depend not only on technical accuracy but also on community acceptance, ethical transparency, and usability.

These challenges can be better understood and systematically examined through established theoretical frameworks. To deepen interpretation of these adoption challenges, the study draws on the Health Belief Model (HBM) and the Technology Acceptance Model (TAM). HBM highlights how perceived susceptibility, benefits, and barriers influence caregivers’ health-related decisions, including ASD help-seeking behaviors [17]. TAM, widely applied in digital health research, explains how perceived usefulness and ease of use shape acceptance of mobile and AI technologies in health care settings [18]. Integrating these frameworks provides a structured basis for examining how caregiver beliefs and provider attitudes interact with system-level constraints to shape adoption of AI-powered ASD screening in Egypt.

Against this backdrop, we conducted a qualitative study in Egypt using focus group discussions (FGDs) and follow-up interviews with health care providers and parents of young children with ASD across diverse urban, semiurban, and rural settings. The primary aim was to explore stakeholder perspectives on the feasibility, acceptability, and perceived ethical and practical enablers and barriers to implementing AI-powered tools for early ASD screening within the Egyptian health system. By situating these perspectives within Egypt’s health system and sociocultural context, and interpreting them through the HBM and TAM, this qualitative inquiry seeks to inform the context-appropriate design and implementation of AI-enabled ASD screening approaches in LMICs. Accordingly, we addressed 2 research questions—research question 1 (RQ1) and research question 2 (RQ2): (RQ1) what barriers and contextual challenges do parents and health care providers identify in current ASD screening, referral, diagnosis, and access-to-support pathways in Egypt? (RQ2) How do parents and health care providers perceive the acceptability and feasibility of AI-powered ASD screening tools, including perceived value, usability, trust, and implementation requirements?

## Methods

### Research Design

This qualitative study used FGDs and individual interviews to explore stakeholder perspectives on AI-assisted autism screening in Egypt. Overall, the study included 49 participants, comprising 28 health care providers (pediatricians, psychologists, family medicine physicians, speech therapists, nurses, and health-information specialists) and 21 parents of children with a confirmed ASD diagnosis aged 6 years or younger. All participants took part in 1 of 7 FGDs, and a subset of these same participants later contributed to brief follow-up individual interviews; no additional participants were recruited solely for interviews. Participants were purposively sampled to reflect diverse professional backgrounds and geographic locations, including urban, semiurban, and rural settings [19]. This diversity facilitated examination of disparities in ASD screening accessibility and cultural perceptions.

FGDs allowed interactive exploration of clinical, cultural, and technical aspects of ASD diagnosis, while individual interviews captured sensitive or nuanced views less likely to emerge in group settings [20,21]. Sessions were conducted separately for health care professionals and parents to minimize power imbalances and encourage open dialogue. Data collection proceeded iteratively alongside early familiarization and preliminary coding; after each FGD (and subsequent follow-up interviews), the facilitation and analysis team conducted rapid debriefs and reviewed emerging codes to assess whether additional sessions were likely to generate substantively new insights. Saturation was judged to have been reached when successive sessions yielded no substantively new codes or theme candidates beyond those already identified and the team agreed that additional data were unlikely to add meaningful conceptual depth [22,23]. Geographic representation was prioritized over equal participant numbers per site.

Participants were recruited through hospitals, specialized ASD clinics, professional networks, autism advocacy groups, special needs schools, and early intervention centers. All received detailed study information and confidentiality assurances and provided written informed consent prior to participation.

A full description of the stepwise thematic analysis process (phases 1-6 of Braun and Clarke’s framework) has been provided in Multimedia Appendix 1.

### Inclusion and Exclusion Criteria

Inclusion criteria were (1) health care professionals directly involved in ASD screening or developmental care, and (2) parents of children with a confirmed ASD diagnosis aged 6 years or younger. Consequently, the realized sample reflects perspectives that are closely anchored in the early detection and screening phase rather than in later, long-term intervention. Parents of children diagnosed during or soon after the preschool years were therefore prioritized, as their reflections most directly inform the feasibility and acceptability of AI-powered early screening tools.

Exclusion criteria were (1) health care providers without direct ASD-related experience, (2) parents of children with suspected but unconfirmed neurodevelopmental disorders, and (3) individuals unwilling to provide consent or unable to complete the full discussion session. This approach ensured that voices from both clinical and caregiving domains were adequately represented, enhancing the relevance and applicability of findings.

### Recruitment Process

Participants were recruited through multiple channels to ensure diversity and relevance. Health care professionals were identified via hospitals, specialized ASD clinics, and professional networks in the selected governorates. Pediatric associations and child development centers facilitated the engagement of pediatricians, psychologists, nurses, and other specialists involved in ASD screening and intervention. All individuals invited agreed to participate, and no invited participants declined or withdrew.

Parents were recruited through autism advocacy groups, special needs schools, early intervention centers, and referrals from pediatric clinics. This ensured inclusion of caregivers with firsthand experience navigating ASD diagnosis and care pathways.

All participants received detailed study information, including objectives, procedures, confidentiality assurances, and their right to withdraw at any time. Written informed consent was obtained prior to participation.

### Geographic Distribution and Representation

The study was conducted across 6 Egyptian governorates selected to capture geographic and demographic diversity, including urban, semiurban, and rural contexts. Governorates were purposively chosen to reflect differences in levels of urbanization, availability of ASD-related services, and health system infrastructure (eg, Ministry of Health and Population primary health care and maternal and child health facilities, university hospitals, and private or nongovernmental centers). This approach ensured that findings reflected a broad spectrum of health care access, cultural beliefs, and diagnostic experiences related to ASD. Rather than aiming for equal sample sizes per site, recruitment focused on meaningful representation of regional disparities in ASD service delivery. Geographic representation was therefore operationalized as maximum-variation coverage of contrasting service environments rather than as proportional sampling based on population size or formal rural–urban quotas.

This geographic diversity, combined with rigorous participant selection, supported a comprehensive understanding of the challenges and opportunities associated with integrating AI-powered ASD screening tools in varied sociocultural and clinical settings.

### Data Collection

Data were collected through semistructured FGDs and individual interviews, combining structured prompts with open-ended dialogue to elicit both guided and spontaneous insights. FGDs lasted 60-90 minutes and were moderated by trained facilitators with backgrounds in public health, ASD, and qualitative research. During parent FGDs, children and, in many cases, the other parent were present in the room so that 1 caregiver could supervise the child while the other participated more actively in the discussion. All facilitators were female members of the research team with prior experience in qualitative interviewing and no prior personal or professional relationship with participants. The same facilitators also conducted the follow-up interviews to ensure continuity and rapport. No repeat interviews were conducted; each participant took part in a single FGD. In addition to the FGDs, short individual interviews were conducted with the same FGD participants to clarify points raised during the group discussion and to allow participants to elaborate on topics they were not comfortable discussing in a group setting. No separate pool of interview participants was recruited; all interviewees had already taken part in an FGD. These interviews were conducted immediately after the FGDs or were scheduled within the same week (as follow-ups for deeper insights). This approach ensured that group-generated themes were accurately understood and provided an opportunity to validate and deepen emerging insights, enhancing the credibility of the analysis.

Given the sensitive nature of ASD screening and emerging AI technologies, moderators received additional training to manage emotionally charged discussions and foster a safe, nonjudgmental environment. To facilitate understanding, participants received a brief explanatory note on AI decision-making, stating:

This allows you to understand the logic behind AI’s decision-making in a simple and clear way; without needing advanced technical knowledge. It’s like AI showing you how it arrived at its decision, so you know it’s not just a random guess.

All individuals invited agreed to participate, and no invited participants declined or withdrew.

### Moderator Training and Power Imbalance Considerations

To address potential power dynamics, FGDs were conducted separately for health care professionals and parents, with moderators trained to foster inclusive participation and encourage open dialogue. Additional individual interviews were conducted to capture sensitive experiences that participants might not have shared in group settings (Multimedia Appendix 1 provides detailed description of moderator training and power-imbalance considerations). All focus groups and individual interviews were conducted by a research team of female public health researchers at the National Research Centre (NRC) with formal training in qualitative interviewing and prior experience in ASD-related services. None had a direct clinical care relationship with the participating families or children, which helped minimize role-related coercion while encouraging open and candid discussion of views on ASD screening and AI tools.

### Discussion Guide Topics

Focus group discussions were guided by 2 core research questions: (RQ1) What barriers and contextual challenges do parents and health care providers identify in current ASD screening, referral, diagnosis, and access-to-support pathways in Egypt? (RQ2) How do parents and health care providers perceive the acceptability and feasibility of AI-powered ASD screening tools, including perceived value, usability, trust, and implementation requirements? The full discussion guide is available in Multimedia Appendix 2.

### Data Recording and Transcription

All FGDs and interviews were audio-recorded and transcribed verbatim. Field notes captured nonverbal cues and group dynamics. Discussions were conducted in Arabic and professionally translated into English to ensure linguistic and conceptual accuracy during thematic analysis. Initial coding was conducted on the original Arabic transcripts by bilingual members of the research team, with English translations used to support team-based analysis and reporting. Arabic and English versions were reviewed side by side, and any discrepancies or ambiguous phrases were resolved through discussion between the coders and the professional translator to preserve the intended meaning. Verbatim transcripts were not formally returned to participants for checking due to resource and logistical constraints; instead, analytic accuracy was supported through verbatim transcription, independent transcript review, peer debriefing, and limited member checking with a purposive subset of participants during follow-up calls to clarify key interpretations.

In reporting quotations, we used anonymized identifiers indicating participant role, gender, and governorate (for example, “Parent-F, Fayoum” and “Pediatrician-M, Cairo”) to illustrate the diversity of perspectives while preserving confidentiality.

### Data Analysis

The analysis followed Braun and Clarke’s (2019) reflexive thematic analysis framework, which involves six iterative phases (1) familiarization with the data through repeated reading of transcripts, (2) generating initial inductive codes, (3) searching for candidate themes by clustering related codes, (4) reviewing and refining themes for coherence, (5) defining and naming themes with operational clarity, and (6) producing the final analytic report integrating quotes and interpretations. NVivo software (version 14; Lumivero) supported coding and data organization. Coding was led by 2 bilingual public health researchers with training in qualitative methods and experience in ASD-related research, supported by a senior qualitative methodologist; all are based at the NRC, and none were involved in direct clinical care of the participating families.

The 2 primary analysts independently read and annotated the transcripts, generated initial inductive codes, and then met regularly to compare and refine the coding frame and candidate theme map, not to calculate interrater reliability but to deepen reflexive engagement with the data, consistent with reflexive thematic analysis. All FGD and interview transcripts were imported into NVivo and analyzed as a single qualitative data corpus using the same inductive coding frame; the data-collection mode (focus group vs individual interview) was retained as a case attribute to enable comparison of patterns across modes when relevant rather than conducting 2 separate analytic processes. For example, codes such as “AI helps nonspecialists,” “AI as first filter,” “AI speeds referrals,” and “AI cannot replace doctors” were clustered into a subtheme labelled “AI as a supportive triage tool,” which was then situated within the broader theme “AI as a supportive tool rather than a replacement for clinicians.”

In line with reflexive thematic analysis, counts (n/N) for parents and health care providers were used descriptively in the tables as signposts of pattern salience; that is, to indicate how many participants contributed to a given code or theme rather than as statistical estimates of prevalence or as the basis for inferential claims [24]. In addition, emergent themes were examined in relation to the HBM and TAM to provide a theoretical lens for interpreting stakeholder perceptions of AI-powered ASD screening. This deductive overlay complemented the inductive analysis, enabling findings to be systematically linked with established models of health behavior and technology adoption.

During analysis, we also explored potential differences in perspectives across health care professional subgroups (pediatricians, nurses, psychologists, and child development specialists); however, given the modest numbers in each subgroup and the substantial overlap in core themes, no systematic divergence in overarching patterns was identified, and themes are therefore reported at the combined “health care provider” level, with subgroup nuances highlighted where relevant. Reflexive journals were maintained by the primary analysts to document assumptions (eg, expectations about the benefits of AI in overstretched public services) and to record how these were challenged by the data; these reflections informed theme naming and interpretation (eg, reframing early “AI will solve gaps” codes as “cautious optimism conditioned by system constraints”). Interpretive rigor was maintained through reflexive team discussions, staged member checking with a purposive subset of participants, peer debriefing with a senior qualitative researcher, and triangulation of perspectives across participant groups. A detailed description of the 6 analytic phases, including examples of coding procedures and reflexivity practices, is provided in Multimedia Appendix 1.

### Ensuring Trustworthiness

This study applied classic criteria of credibility, transferability, dependability, and confirmability [25-27]. Credibility was established through triangulation of FGDs and interviews, member checking, and peer debriefing. Member checking occurred at 2 stages. First, at the end of each focus group and interview, moderators briefly summarized the main discussion points and explicitly invited participants to correct, nuance, or add to these summaries, allowing immediate verification of the researchers’ understanding. Second, after preliminary theme development, the research team prepared concise written summaries of the key themes and a small set of illustrative quotes and contacted a purposive subset of parent and clinician participants from different governorates (via brief follow-up calls or messaging apps) to review these summaries. Participants were asked whether the themes and examples reflected their experiences, and their feedback was used to refine thematic boundaries, adjust wording of themes, and address any apparent misinterpretations. Formal transcript-based member checking (returning full transcripts to all participants) was not undertaken due to resource and logistical constraints; however, this staged, selective approach allowed verification of core interpretations without recontacting every participant or overburdening busy clinicians and caregivers.

Transferability was supported by thick descriptions of participant experiences, service settings, and contextual factors across diverse regions, enabling readers to assess how findings may apply to similar LMIC contexts. Dependability was ensured by documenting analytic procedures and maintaining an audit trail. The audit trail included decision logs on coding, theme development, and changes to the analytic framework over time. Confirmability was strengthened through reflexivity and integration of direct participant quotes into findings. The primary analysts maintained reflexive journals to document their assumptions and how these were challenged by the data; these reflections were discussed in team meetings and peer debriefing sessions with a senior qualitative researcher, supporting critical examination of alternative interpretations and reducing the risk of unchecked researcher bias. Triangulation across data sources (FGDs and follow-up interviews) and participant groups (parents and health care providers) further contributed to the credibility and confirmability of the analysis. Details of the trustworthiness measures are provided in Multimedia Appendix 1.

### Ethical Considerations

Ethical approval for this study was granted by the NRC of Egypt (approval number 2705250141). All participants provided written informed consent prior to participation. Before participation, individuals received detailed study information, including objectives, procedures, confidentiality measures, and their right to withdraw at any stage without justification or consequences. Participants had ample time to review consent forms, ask questions, and opt out if desired. To protect privacy, all data were anonymized, securely stored, and accessible only to authorized researchers. Participants received reimbursement for travel costs and a modest goodwill payment of US $20, as approved by the NRC ethical committee and funded by Ulster University (cost center 71799); these tokens were intended to offset participation-related expenses rather than act as coercive incentives. Concerns raised by participants were incorporated into the analysis to highlight ethical implications of AI integration in screening of neurodiversity disorders [28,29]. Researchers followed international ethical guidelines, including the Declaration of Helsinki and General Data Protection Regulation data protection standards [30].

Through comprehensive consent procedures, strict confidentiality measures, and reflexive ethical discussions, this study upheld responsible and ethical qualitative research practices. Participation was fully voluntary, and participants were informed that they could withdraw at any time without consequences. Data were collected in a confidential manner, deidentified and delinked from personal identifiers, and stored in a secure location. In addition, data collection and handling complied with Egypt’s personal data protection framework; identifiable information was stored on password-protected institutional servers at the NRC in Cairo, with access restricted to the core research team, and deidentified analytic datasets were stored separately and shared only in encrypted form. Information disclosure (“making sure participants understand”) was guaranteed according to the recommendations of prior work on Egyptian patients’ and guardians’ perceptions of clinical informed consent [31].

This study was designed, conducted, and reported in accordance with the COREQ (Consolidated Criteria for Reporting Qualitative Research), and a completed COREQ checklist is provided in Multimedia Appendix 3.

## Results

### Overview

Table 1 summarizes the composition of the study participants, including parents and health care providers, across 6 Egyptian governorates. Participants were recruited from governorates representing diverse geographic and sociodevelopmental settings across Egypt, including urban (Cairo), urban/semiurban (Giza and Damietta), mixed urban–rural (Dakahlia and Assuit), and predominantly rural/semiurban (Fayoum). The sample comprised clinicians (pediatricians, psychologists, family medicine physicians, speech therapists, nurses, and health-information specialists) and parents of children with diagnosed ASD. Participant numbers varied by site depending on service availability and community size rather than equal quotas per governorate, consistent with qualitative sampling principles.

**Table 1:** Participant description (N=49) by geographic location, professional role, and participant type.

Governorate/region [32-36]	Setting type	Parents, n/N	Health care providers, n/N	Health care provider roles
Dakahlia (Lower Egypt, mixed urban–rural)	Autism centers, outpatient clinics	3/21	4/28	Pediatrician, psychologist, head nurse, and family medicine physician
Cairo (cities, urban)	MCH^a^ centers	5/21	7/28	Pediatricians, 2 psychologists, head nurse, 2 general practitioners, and health-information specialists
Giza (cities, urban/semiurban)	Early screening units	3/21	3/28	Pediatrician, speech therapist, and MOHP^b^ officer
Fayoum (Upper Egypt, rural/semiurban)	Primary health units	5/21	6/28	Pediatrician, 2 psychologists, head nurse, PHC^c^ physician, and health-information specialist
Damietta (coastal, urban/semiurban)	Pediatric clinics, NGO^d^	2/21	4/28	Pediatrician, 2 psychologists, and speech therapist
Assuit (Upper Egypt, mixed urban–rural)	Community health centers	3/21	4/28	Pediatrician, psychologist, head nurse, and PHC Physician

^a^MCH: maternal and child health care.

^b^MOHP: Ministry of Health and Population.

^c^PHC: primary health care.

^d^NGO: nongovernmental organization.

In the main thematic tables (Tables 2-6), we therefore report absolute counts with explicit denominators for each stakeholder group (for example, 20/21 parents; 25/28 health care providers) rather than percentages, to reflect the qualitative, nonstatistical nature of the data while still allowing readers to assess the salience of each view across participants [24,37].

**Table 2:** Theme 1: reported challenges in autism diagnosis and pathways, mention counts (n/N) by participant group.

Challenge^a^	Parents, n/N	Health care providers, n/N
Lack of ASD^b^-specific training for health care professionals	18/21	21/28
No standardized ASD screening protocols	13/21	16/28

^a^Values represent the number of participants (n) who spontaneously mentioned each challenge. Denominators (N) refer to total participants in each group (parents=21 and health care providers=28). These counts indicate thematic salience, not prevalence or consensus. Illustrative quotations capture both typical and divergent views to reflect the full spectrum of perspectives and should not be interpreted as statistical estimates.

^b^ASD: autism spectrum disorder.

**Table 3:** Theme 2: limited awareness, social stigma, and misconceptions about autism, mention counts (n/N) among parents.

Challenge^a^	Parents, n/N
Stigma and fear of judgment	11/21
Misconceptions (autism as temporary or due to poor parenting)	8/21
Lack of parental awareness about ASD^b^ screening	7/21

^a^Values represent the number of participants (n) who spontaneously mentioned each challenge. Denominators (N) refer to total participants in each group (parents=21). These counts indicate thematic salience, not prevalence or consensus. Illustrative quotations capture both typical and divergent views to reflect the full spectrum of perspectives and should not be interpreted as statistical estimates.

^b^ASD: autism spectrum disorder.

**Table 4:** Theme 3: reported barriers to accessing autism diagnostic and support services: mention counts (n/N) by participant group.

Barriers^a^	Parents, n/N	Health care providers, n/N
Shortage of ASD^b^ specialists in local areas	13/21	23/28
Long waiting times for appointments	12/21	11/28
High cost of private consultations	9/21	11/28
Travel distance/time burden	15/21	10/28

^a^Values represent the number of participants (n) who spontaneously mentioned each barrier. Denominators (N) refer to total participants in each group (parents=21 and health care providers=28). These counts indicate thematic salience, not prevalence or consensus. Illustrative quotations capture both typical and divergent views to reflect the full spectrum of perspectives and should not be interpreted as statistical estimates.

^b^ASD: autism spectrum disorder.

**Table 5:** Theme 4: perceived role of artificial intelligence (AI) in autism screening, mention counts (n/N) by participant group.

Factor^a^	Parents, n/N	Health care providers, n/N
Support for AI-based screening	9/21	19/28
Need for culturally adapted tools	12/21	16/28
Preference for explainable AI-generated results	15/21	17/28

^a^Values represent the number of participants (n) who spontaneously mentioned each factor. Denominators (N) refer to total participants in each group (parents=21 and health care providers=28). These counts indicate thematic salience, not prevalence or consensus. Illustrative quotations capture both typical and divergent views to reflect the full spectrum of perspectives. Figures should not be interpreted as statistical estimates.

**Table 6:** Theme 5: reported concerns about artificial intelligence (AI) adoption, mention counts (n/N) by participant group.

Concern^a^	Parents, n/N	Health care providers, n/N
Data privacy and security risks	17/21	23/28
Fear of AI replacing human judgment	6/21	—^b^
Limited digital literacy among parents	4/21	—^b^
User-friendly interface for non-specialist health care providers	—^b^	21/28

^a^Values represent the number of participants (n) who spontaneously mentioned each concern. Denominators (N) refer to total participants in each group (parents: 21; health care providers: 28). These counts indicate thematic salience, not prevalence or consensus. Illustrative quotations capture both typical and divergent views to reflect the full spectrum of perspectives. Figures should not be interpreted as statistical estimates.

^b^Not applicable.

This analysis drew upon 7 FGDs involving key health care providers, health professionals, and parents of children with ASD across 6 governorates in Egypt. Each group included approximately 7 participants, totaling 28 health care professionals and 21 parents. Health care professionals included heads of departments for children with special needs, chief technicians, physicians, nurses, speech therapists, and health-information specialists. The mean age of health care providers was 43.3 (SD 11.0) years. The mean age of parents was 44.3 (SD 10.6) years. Among their children, 66.7% (14/21) were male and 33.3% (7/21) female, with a mean age at diagnosis of 2.2 (SD 0.6) years; 80.9% (17/21) were classified as having moderate autism. Educational attainment varied across groups: among health care providers, 39.3% (11/28) held graduate degrees, 32.1% (9/28) postgraduate qualifications, and 28.6% (8/28) technical education. Among parents, 57.1% (12/21) held graduate degrees, 23.8% (5/21) postgraduate qualifications, and 19.1% (4/21) secondary or technical education.

Thematic analysis of the focus group and interview data identified 5 major themes that captured stakeholder perspectives on the feasibility, acceptability, and challenges of AI-powered autism screening in Egypt. Absolute counts with denominators (n/N) are presented to indicate how many participants in each group (parents and health care providers) raised each issue during the discussions. Importantly, these figures should be interpreted as indicators of thematic salience rather than statistical prevalence or consensus, given the exploratory qualitative design and the fact that not all participants were asked identical questions in a standardized format.

To reinforce this, table footnotes clarify that counts (n/N) reflect how frequently topics arose rather than the degree of agreement across the sample. While the abstract summarizes the 5 overarching themes conceptually according to AI adoption factors, the following Results section presents them in the empirical order in which they emerged during thematic analysis.

These figures should be viewed as indicators of thematic salience rather than measures of agreement or prevalence, as not all participants were asked identical questions in a standardized format. Instead, they reflect how frequently particular topics arose during the conversations and the relative emphasis placed on them by participants. To ensure analytic rigor, the interpretation of these findings was informed by reflexive consideration of the research team’s positionality, clinical experience, and prior exposure to autism-related work in Egypt. The 5 themes emerging from the analysis were delays in autism diagnosis and inconsistent pathways; limited awareness, social stigma, and misconceptions about autism; barriers to accessing diagnostic and support services; perceived role of AI in autism screening; and balancing trust, transparency, and data protection in AI screening.

Tables 2-6 summarize the 5 major themes derived from this analysis. Each table presents key subthemes, absolute counts with denominators for parents and health care providers, representative participant quotations, and brief interpretive notes. This structure facilitates comparison across themes while preserving a clear link between each table and its corresponding narrative subsection in the Results. Themes 1-3 primarily address RQ1 by characterizing current pathway-related and access-related barriers to timely ASD screening and support, whereas Themes 4 and 5 primarily address RQ2 by examining perceived acceptability, feasibility, and trust-related considerations for AI-enabled ASD screening.

### Theme 1: Delays in Autism Diagnosis and Inconsistent Pathways

In relation to RQ1 (current pathway-related barriers), most health care providers reported lacking specific training in ASD diagnosis, and many raised concerns about the absence of standardized screening protocols. These delays were most often attributed to long waiting lists, limited specialist availability, and the absence of standardized referral pathways. Diagnostic inconsistency was another recurring concern, with many parents and more than half of health care professionals noting that children often received conflicting assessments from different providers.

One parent described the protracted wait for assessment:

I had to wait over six months for my child to be assessed, and even then, I wasn’t sure if the diagnosis was correct because different doctors gave me different answers.

Parent-M, Cairo

This quote illustrates 2 critical barriers: significant delays due to workforce shortages and diagnostic inconsistency arising from the absence of standardized screening protocols. Together, these factors contribute to late interventions and parental uncertainty.

Health care professionals echoed these concerns, with one psychologist noting:

We see many children after they have been to three or four different doctors, each giving a different opinion.

Psychologist-F, Assuit

Parents reported long diagnostic delays:

I went to three doctors before someone said autism” (Parent, Fayoum)—while healthcare providers cited limited resources and high caseloads as major obstacles. Healthcare provider described “a system that reacts late, not one that screens early.

Speech therapist-M, Damietta

This reflects fragmentation of care pathways and highlights why nearly two-thirds of parents and half of health care providers in our sample emphasized the urgent need for standardized assessment tools. Importantly, participants also drew connections between these diagnostic inconsistencies and later discussions of AI, suggesting that standardized digital tools could harmonize assessments and reduce conflicting diagnoses (Theme 5).

Transitioning from diagnostic delays, participants also described how broader societal perceptions of autism further compound these challenges, as explored in Theme 2.

### Theme 2: Limited Awareness, Social Stigma, and Misconceptions About Autism

Cultural stigma and misinformation emerged as significant barriers to early ASD diagnosis and were raised by many parents. Misconceptions, such as autism being temporary or caused by poor parenting, were frequently reported, and several parents were unaware that early screening was even possible.

Parents described community-level misunderstandings, such as attributing autism symptoms to poor parenting, stubbornness, or spiritual causes.

People told me my child was just naughty, or that I was spoiling him too much.

Parent-F, Assuit

This highlights the community-level stigma faced by more than half of parents in the sample, reinforcing how misconceptions delay help-seeking behavior. Health care professionals also noted that misconceptions among general practitioners sometimes led to misdiagnosis, resulting in children being referred to speech therapy or psychiatry without autism-specific assessment.

A pediatrician from Fayoum explained:

Some doctors still think autism means a child cannot speak at all, so they miss milder cases.

Pediatrician-M, Fayoum

By linking parental stigma with professional misconceptions, participants emphasized that awareness deficits are multilayered, spanning families, communities, and frontline medical providers. These deficits not only prolong diagnostic delays but also shape the systemic barriers discussed in Theme 3. In particular, stigma and misconceptions were seen as amplifying existing access problems, since families already hesitant to seek help often face structural hurdles once they do attempt to engage with services.

### Theme 3: Barriers to Accessing Diagnostic and Support Services

Whereas Theme 2 captured attitudinal and cultural barriers to early ASD identification, Theme 3 focuses on structural and logistical constraints that hinder access to timely diagnosis and ongoing support. Barriers to service access were reported by many parents and most health care providers. These included long travel distances, high service costs, and the scarcity of autism-specialized centers, particularly in rural areas.

One mother from Fayoum explained:

The nearest centre is two hours away, and I cannot afford to travel every week.

Parent-F, Fayoum

Health care providers recognized these structural barriers. A psychologist from Cairo noted:

Families in rural areas have very limited options. Sometimes we try to offer online consultations, but internet quality is a problem.

Psychologist-M, Cairo

Financial constraints were also significant. One parent shared:

Therapy costs a lot, and we have to choose between paying for treatment or other basic needs.

Parent-M, Dakahlia

These findings underline the intersection of geographic, economic, and infrastructural barriers, which can delay both diagnosis and intervention. This was particularly salient among parents and was also frequently highlighted by health care providers, underscoring the disproportionate burden borne by families navigating multiple constraints simultaneously. Against this backdrop of limited access, many participants suggested that AI tools could potentially bridge these gaps by offering more accessible first-line screening, a possibility explored further in Theme 4. When discussing digital health options, parents and providers repeatedly pointed out that limited internet access and affordability challenges could constrain the feasibility of AI-powered tools even as they recognized their potential benefits.

### Theme 4: Perceived Role of AI in Autism Screening

In relation to RQ2 (acceptability and feasibility of AI-enabled screening), optimism about AI-powered tools was expressed by many health care professionals and a substantial proportion of parents, who emphasized the potential for early risk identification in underserved areas. Some pediatricians and child development specialists highlighted AI’s promise for accelerating referrals and supporting overburdened systems, while frontline nurses and primary care physicians more often focused on how such tools might fit within existing workloads and communication with families.

A pediatrician in Giza stated:

AI could help us identify at-risk children sooner, so we can refer them before the delay becomes too long.

Pediatrician-F, Giza

Parents similarly expressed cautious optimism:

If it’s accurate and easy to use, I would try it for my child.

Parent-F, Assuit

Most parents viewed AI as a tool that could support earlier detection and broaden access to assessment, particularly in areas with limited specialist services:

If the app can tell me early that my child is at risk, I can go to the doctor sooner instead of waiting for years.

Parent-F, Fayoum

However, a minority expressed discomfort with technology-mediated interactions, preferring face-to-face communication:

I prefer someone to talk to me directly, not an app that gives me a score.

Parent-M, Cairo

These divergent views highlight the need for AI screening tools to be positioned as supportive aids rather than replacements for human care. Overall, enthusiasm was clear, but participants emphasized that meaningful adoption would require addressing structural barriers (Theme 3) and building trust through transparency and usability (Theme 5).

### Theme 5: Balancing Trust, Transparency, and Data Protection in AI Screening

Concerns about trust and usability emerged strongly, with many parents and health care providers citing data privacy as their foremost worry. Psychologists often linked these concerns to existing stigma and the risk of labeling, whereas pediatricians tended to emphasize clinical responsibility for acting on AI-generated risk scores. Participants also frequently emphasized the importance of clear, understandable outputs, and some parents expressed concern that AI might replace human judgment.

One pediatrician stressed the importance of maintaining human oversight:

AI should not replace a specialist. A diagnosis should come from a doctor, not an algorithm.

Pediatrician-F, Dakahlia

Parents voiced privacy concerns:

If I use an app to screen my child, how do I know my data won’t be misused? I don’t want personal information shared without my consent.

Parent-M, Damietta

Digital literacy emerged as an additional barrier, especially in rural contexts:

I am not good with technology. If the AI screening tool is too complicated, I won’t know how to use it properly for my child.

Parent-F, Fayoum

Health care providers also highlighted usability concerns. A physician explained that:

AI screening tools must be easy to use, especially for general practitioners and nurses who are not ASD specialists.

PHC Physician-F, Assuit

A nurse echoed this point, adding:

If the system is too complicated, healthcare providers won’t use it, and parents will struggle to understand the results.

Head Nurse-F, Cairo

However, a few younger health care providers expressed optimism about automation:

If AI can handle screening, we can focus on therapy and follow-up.

Speech therapist-M, Damietta

This generational contrast reflects differing comfort levels with digital integration in clinical care. Across all sites, participants linked AI adoption to issues of fairness, privacy, and equity.

One policymaker warned:

Technology must not widen the gap between rich and poor families.

MOH officer-F, Giza

Many parents wanted assurances of data protection, with one noting:

Once data is uploaded, we lose control. That scares us.

Parent-M, Fayoum

In contrast, a few tech-savvy participants felt that:

Data collection is normal—everything is online now.

Technology specialist-F, Cairo

Their comfort with digital data contrasts with the majority’s apprehension, illustrating generational and digital literacy divides. To accommodate both digitally inexperienced parents and nonspecialist providers, participants underscored the need for intuitive design. Trust in AI was therefore linked not only to accuracy and cultural fit but also to privacy protections, clarity of outputs, and user-friendliness.

This final theme loops back to Theme 1’s concern with diagnostic inconsistencies, reinforcing that AI adoption could help standardize pathways if implemented with strong privacy protections and clear communication.

**Figure 1:**
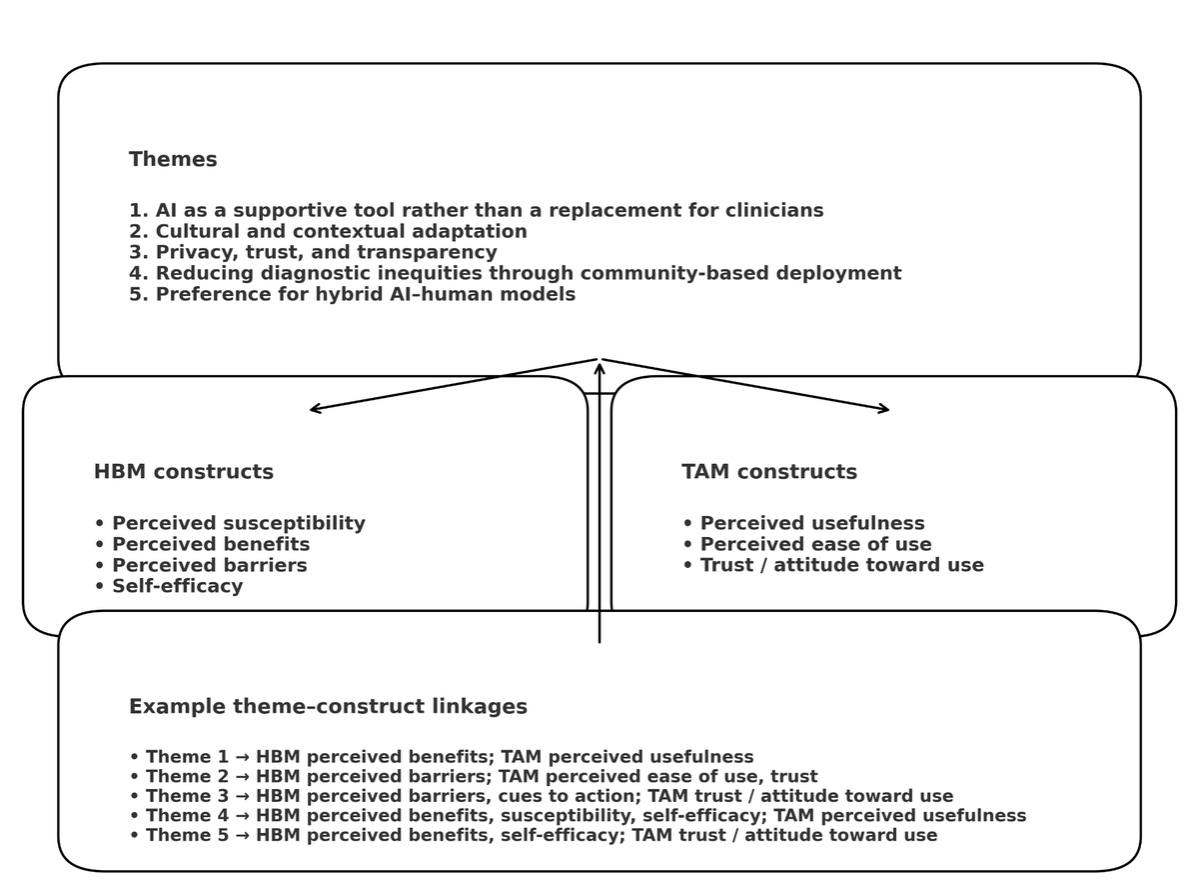
Conceptual mapping of emergent themes to Health Belief Model (HBM) and Technology Acceptance Model (TAM) constructs.

In addition to the 5 major themes, several divergent perspectives emerged. For example, while many parents emphasized accessibility as a key advantage, some questioned whether AI tools might replace human empathy. Similarly, a minority of clinicians expressed optimism about algorithmic decision support, contrasting with the dominant skepticism. These variations illustrate the heterogeneity of experiences and highlight areas for tailored implementation strategies. To support interpretation, the 5 themes are also conceptually mapped onto HBM and TAM constructs; this integration is summarized in Figure 1 and elaborated in the Discussion.

## Discussion

### Overview

This study explored stakeholder perspectives on the feasibility, acceptability, and perceived ethical and practical enablers and barriers to implementing AI-powered tools for early ASD screening within the Egyptian health system. Overall, participants recognized that AI-powered tools could help bridge diagnostic gaps, support earlier detection, and extend services to underserved areas. At the same time, they highlighted substantial barriers that must be addressed for successful adoption, including systemic delays and fragmented care pathways, lack of standardized diagnostic practices, pervasive stigma and low awareness, concerns about cultural appropriateness and transparency of AI decisions, and limitations in digital infrastructure and workforce training.

As illustrated in Figure 1, the themes align closely with core constructs of the HBM and TAM. Perceptions of AI as a supportive triage tool, particularly for nonspecialists in underserved areas, reflect strong perceived benefits and perceived usefulness, which are key drivers of screening uptake in HBM- and TAM-informed frameworks. In contrast, concerns about data privacy, algorithmic opacity, and cultural mismatch map onto perceived barriers, perceived ease of use, and trust-related TAM constructs and help explain ambivalence despite overall cautious optimism. The preference for hybrid AI–human models illustrates how trust, self-efficacy, and attitudes toward use are negotiated in practice. Stakeholders were more comfortable when AI outputs were explicitly embedded within human oversight and relational care. These relationships are summarized in the conceptual mapping presented in Figure 1 and highlight potential leverage points for implementation, including strengthening perceived benefits and usefulness, reducing perceived barriers, and actively building trust and self-efficacy around AI tools.

Viewed through this HBM/TAM lens, the findings highlight both the promise and limitations associated with AI-driven neurodiversity screening. Consistent with prior research, AI-based screening tools offer a scalable, accessible, and cost-effective solution to bridging gaps in ASD diagnosis, particularly in underserved regions [13,38]. In keeping with Braun and Clarke’s [24] call for thematic analysis to move beyond description toward theoretically informed interpretation, our findings link participants’ accounts to established constructs in health behavior and technology adoption frameworks. However, stakeholder concerns regarding algorithmic bias, data privacy, and ethical implications suggest that AI models must be rigorously validated and co-designed with input from health care professionals, caregivers, and neurodivergent individuals [39,40]. Our emphasis on culturally sensitive AI models therefore reflects a call for AI tools to follow and extend the same principles of careful translation, local validation, and reflexive use that are increasingly expected of human-administered instruments, rather than holding AI to a fundamentally different standard [16,28,29,41-48].

Viewed in a broader context, our findings both converge with and extend international evidence on AI-enabled and digital approaches to autism and neurodevelopmental screening. Studies from high-income countries have similarly reported cautious optimism about AI and mobile tools, alongside concerns regarding algorithmic transparency, data privacy, and the risk of depersonalized care [9,29,49,50]. Our participants’ preference for hybrid AI–human models echoes this literature, which consistently emphasizes the importance of human oversight and relational communication when delivering AI-generated outputs [9,28,40,51]. By bringing these strands together, this study contributes a LMIC-based perspective that both confirms global concerns and foregrounds context-specific implementation challenges [15,16,47,48].

From a reflexive standpoint, the research team’s professional backgrounds in public health, autism screening, and health care systems may have sensitized the analysis toward issues of access, policy implications, and sociocultural context. This positionality informed both theme construction and interpretation, particularly in highlighting equity considerations for rural and underserved groups. Explicitly acknowledging positionality strengthens the transparency of analytic choices, in line with qualitative rigor guidelines [52].

AI tools have demonstrated high accuracy in speech pattern recognition, facial analysis, and behavioral assessments, improving early-stage ASD risk detection [9]. Consistent with earlier research, this study supports a hybrid approach, where AI functions as a decision-support tool rather than a diagnostic substitute [41,42,51].

### AI Skepticism vs Parental Trust: Divergent Stakeholder Perspectives

One of the study’s key findings is the contrast between health care professionals’ AI skepticism and parents’ greater openness to AI-driven screening. Many parents in the study viewed AI as a potential tool for early intervention, particularly in regions with limited specialist access. As one parent noted, “If it can tell me early that something is wrong, I don’t care if it’s a machine; as long as I can get help faster.” This quote illustrates how parents interpret AI’s value pragmatically, privileging speed and access over abstract concerns about reliability.

Conversely, many health care professionals in this study questioned AI’s reliability, highlighting concerns that machine learning models might misinterpret symptoms, particularly for nonverbal children or those with atypical ASD presentations. This skepticism reflects broader concerns about algorithmic opacity and AI’s inability to capture the complexity of neurodivergent traits [40,53-55].

### Ethical Considerations in AI Adoption for ASD Screening

The integration of AI-driven screening tools raises pressing ethical concerns that must be addressed for widespread adoption. One major challenge is algorithmic bias, as many models are trained on Western datasets, limiting their applicability to diverse global settings [15]. This is particularly relevant in Egypt, where sociocultural differences in autism perception and diagnosis criteria may not align with Western-centric models. Participants’ concerns mirror broader debates emphasizing the importance of cultural adaptation [47].

Data privacy emerged as another primary concern. Trust was closely tied to transparency in data governance and safeguards [40,51].

Finally, participants worried about misdiagnosis and emotional distress—false positives producing anxiety and false negatives delaying intervention. These dual anxieties underscore that participants’ accounts should be understood not as statistical generalizations but as socially constructed experiences of risk and uncertainty [46].

### Bridging Urban vs Rural Disparities in ASD Diagnosis

A key contribution of this study is its comparative analysis of urban and rural ASD screening barriers. While urban parents and health care professionals reported long waiting times and high costs as primary challenges, rural participants highlighted severe shortages of ASD specialists and a widespread lack of awareness about screening tools. These findings align with prior research on health care disparities in low-resource settings, which suggests that AI-powered screening must be adapted to infrastructural limitations and literacy levels of diverse communities [8,43].

### Theoretical Contributions: Behavior Change, Trust, and Stigma

This study contributes to theoretical frameworks explaining health technology adoption and behavior change in autism screening. Consistent with the HBM, parental decisions regarding AI-powered screening tools were shaped by their perceptions of susceptibility to ASD, the benefits of early detection, and concerns about potential barriers particularly stigma and data misuse [56]. Participants’ concerns about stigma align with Goffman’s theory of social labeling, where fear of judgment discourages disclosure [57].

Furthermore, trust-based adoption models and the TAM were evident in health care professionals’ skepticism and parents’ mixed acceptance driven by concerns over ease of use, usefulness, and trust in AI-generated outputs [55,58,59].

### Policy Recommendations With Implementation Strategies

Building on these empirical and theoretical insights, several practical and policy-level recommendations can be made to support the ethical and effective implementation of AI-driven ASD screening in Egypt and similar contexts.

AI tools should be trained and validated on diverse datasets that reflect the cultural, linguistic, and behavioral characteristics of Egyptian children [13,15,16,39,49,50]. AI systems can follow the same approach by using locally collected training data, documenting the provenance and limitations of datasets, and involving Egyptian clinicians, caregivers, and autistic individuals in co-design and evaluation [16,28,29,47-49]. Such steps would help ensure that AI does not simply reproduce Western diagnostic assumptions but is genuinely responsive to local sociocultural realities [15,16,28,29,48,49].

AI adoption must be underpinned by national legal frameworks that regulate data storage, privacy, and consent. Without such frameworks, participants’ anxieties about surveillance and discrimination could limit uptake, as also reported in other LMIC contexts [15].

Participants emphasized that parents’ trust depends on transparent communication regarding how AI screening tools operate, where data are stored, and who has access. Transparent governance and communication have been shown to improve uptake of AI-based health tools [51].

A recurring theme was the value of combining AI outputs with human judgment. Parents and health care providers agreed that AI should assist, not replace, clinicians. This aligns with global guidance emphasizing AI as an augmentative tool [9], and reflects participants’ preference for “shared accountability” between humans and technology.

To address diagnostic inequities, AI-enabled screening should be deployed in primary care facilities and community health centers in rural areas. Participants in rural settings described stigma, lack of awareness, and absence of specialists as barriers, reinforcing the importance of deploying accessible, low-literacy screening tools. Training community health providers to use AI tools could decentralize early detection and improve referral pathways. This approach mirrors other successful models of digital health integration in low-resource contexts [54].

Successful AI adoption will depend not only on clinical readiness but also on public understanding and digital competence. Parents and caregivers expressed both curiosity and apprehension about AI, underscoring the need for structured awareness campaigns. Digital literacy training has been shown to improve uptake of AI tools in LMIC health care settings [47].

Stigma remains a powerful deterrent to early ASD diagnosis. Participants’ accounts revealed that some families avoid clinics out of fear of labeling, echoing prior national studies on autism stigma in Egypt [8].

Health care providers and frontline staff require training not only in the technical use of AI tools but also in communicating AI results to families with empathy and sensitivity. Participants stressed the risk of “cold delivery” of AI outputs; thus, training must emphasize the human element of care to prevent alienation. Building capacity in both technical literacy and interpersonal skills will ensure AI integration enhances, rather than undermines, the therapeutic alliance between families and health care systems.

### Strengths

This study is among the first to explore stakeholder perspectives on AI-powered autism screening in Egypt, offering valuable insights for the development and implementation of such tools in LMICs. The use of reflexive thematic analysis allowed for the identification of context-specific enablers and barriers, drawing from the lived experiences of both health care professionals and parents. Purposive sampling ensured that participants represented a wide range of geographic and professional backgrounds, increasing the diversity of perspectives captured. Together, these design choices support conceptual transferability to other LMIC settings facing similar diagnostic and infrastructural challenges. All parent participants had children aged 6 years or younger, anchoring parental perspectives in early detection contexts.

### Limitations

As with many qualitative designs, findings are context-bound and not intended for statistical generalization. Instead, the aim was to generate nuanced, situated understandings that may have conceptual transferability to similar LMIC contexts. Although the sample achieved geographic variation, the number of FGDs was necessarily constrained by logistical and resource limitations, which shaped the scope of representation. The sample was not designed to be proportionate to population distributions across urban and rural areas and was not powered for formal statistical comparisons between geographic strata; rather, rural and urban participants were included to surface key contextual patterns and implementation considerations. As discussed above, the research team’s positionalities, including professional experience in public health, autism research, and digital health, may have influenced data interpretation, particularly in prioritizing equity and access themes; this reflexive awareness was explicitly documented and used to critically interrogate analytic decisions.

Although all individuals who were invited agreed to participate, which may reflect greater interest or comfort with discussing AI and autism, we attempted to mitigate this potential selection bias by recruiting across multiple governorates, facility types, and professional roles to capture a wide range of perspectives. However, socioeconomic position was not systematically measured or analyzed, and any socioeconomic variation in the sample is therefore inferred indirectly through differences in governorate and service setting rather than examined explicitly. In addition, because all participating parents had children aged 6 years or younger, perspectives related to later intervention phases were not represented, and we were not able to systematically compare views on early detection vs long-term intervention. Similarly, the numbers of participants within individual health care professional subgroups were not sufficient to support robust within-profession comparisons, and the analysis was therefore conducted at the combined health care provider level, with any role-specific nuances interpreted cautiously.

Additionally, while separate FGDs for health care professionals and parents minimized power imbalances, the group setting may have led to social desirability bias, with participants potentially moderating their comments in line with perceived group norms. The presence of AI-related stigma and limited public familiarity with AI screening could also have influenced the openness of responses, despite measures taken to build rapport. Future research could incorporate longitudinal designs and follow-up interviews to explore how attitudes evolve as AI screening tools are introduced and piloted in local health care systems.

These limitations do not undermine the validity of the findings but rather frame them as contextually situated insights, highlighting the value of qualitative inquiry for shaping ethical and culturally sensitive AI interventions in LMICs.

### Conclusion

This study highlighted how feasibility, acceptability, and perceived ethical and practical enablers and barriers shape stakeholder attitudes toward implementing AI-powered tools for early ASD screening within the Egyptian health system. While parents were generally open to AI tools for early detection, health care professionals expressed caution, emphasizing the need for rigorous validation, culturally adapted algorithms, and integration within existing diagnostic pathways.

AI-powered tools hold significant promise for addressing diagnostic delays and specialist shortages in Egypt and other LMICs. However, their success depends on transparent governance, stakeholder engagement in co-design, and human oversight to safeguard against bias and misuse.

By integrating technological innovation with culturally sensitive implementation strategies, AI-powered screening tools could contribute to more equitable, timely, and accurate autism identification. In practical terms, this entails embedding AI within hybrid human–AI care models, aligning governance frameworks with community expectations and ethical standards, and sustaining engagement with families and clinicians to ensure that AI enhances, rather than replaces, human-centered care.

## Data Availability

The datasets used and/or analyzed for the current study are anonymous and are available from the corresponding author on request.

## Appendix

Multimedia Appendix 1 Expanded methodological details.

Multimedia Appendix 2 Focus group and interview discussion guide.

Multimedia Appendix 3 COREQ (Consolidated Criteria for Reporting Qualitative Research) checklist.

## Abbreviations

AI: artificial intelligence
ASD: autism spectrum disorder
COREQ: Consolidated Criteria for Reporting Qualitative Research
FGD: focus group discussion
HBM: Health Belief Model
LMIC: low- and middle-income country
NRC: National Research Centre
RQ1: research question 1
RQ2: research question 2
TAM: Technology Acceptance Model
