# Supplementary material for: Feasibility and Acceptability of AI-Powered Tools for Early Autism Screening in Egypt: Semistructured Focus Group Study
Source: J Med Internet Res. 2026 Apr 7;28:e82564. doi: 10.2196/82564 (PMC13148128; doi:10.2196/82564)
Supplement: Multimedia Appendix 1 [file jmir_v28i1e82564_app1.docx]

**Multimedia Appendix 1**

**Expanded Methodological Details**

**Moderator Training and Power Imbalance Considerations**

To ensure inclusive and equitable participation, moderators underwent training in managing sensitive discussions on autism spectrum disorder (ASD) and emerging digital health tools. Training emphasized strategies for:

- Creating a safe, nonjudgmental atmosphere during focus group discussions (FGDs).
- Addressing **potential power imbalances** between healthcare professionals and parents by conducting sessions separately.
- Using **inclusive facilitation techniques** such as round-robin participation, structured turn-taking, and probing questions to ensure contributions from all participants, including quieter voices.
- Managing emotionally charged discussions with empathy while maintaining focus on study objectives.

All moderators were females. Individual interviews complemented FGDs by capturing highly personal or sensitive perspectives that participants may not have felt comfortable sharing in group settings. This dual approach enhanced the breadth and depth of perspectives collected.

**Data Analysis**

**Reflexive Thematic Analysis: Six-Phase Workflow**

The data analysis followed Braun and Clarke’s (2019) Reflexive Thematic Analysis framework, ensuring systematic, transparent, and reflexive interpretation. The six phases were applied as follows:

**Phase 1: Data Familiarisation**

All focus group discussions and interviews were transcribed verbatim. The research team engaged in repeated, immersive readings of the transcripts to become deeply familiar with the content. Audio recordings were reviewed alongside the transcripts to ensure accuracy, and reflective memos were maintained to capture initial impressions, potential patterns, and contextual insights. Each transcript was read in full a minimum of three times by each researcher, with the first reading focusing on overall content, the second on noting salient phrases or recurrent ideas, and the third on refining these notes into preliminary analytical observations. Reflexivity was actively maintained through journaling, where researchers documented their assumptions, positionality, and emotional responses to the data to minimise bias and enhance interpretative depth. To mitigate potential bias, regular discussions were held with all researchers from developmental psychology, public health and sociology backgrounds, ensuring that different disciplinary perspectives were integrated into interpretation. These notes served as an early analytic resource during later coding and theme development stages. This reflexive practice helped the team remain aware of how professional backgrounds, cultural expectations, and personal assumptions could shape theme construction.

Participants were not asked to provide feedback on the preliminary findings due to time and logistical constraints. Instead, rigor was enhanced through peer debriefing, triangulation across participant groups (parents and clinicians), and involvement of multiple coders to validate interpretations.

**Phase 2: Generating Initial Codes**

Coding was conducted inductively, allowing themes to emerge from the dataset rather than from a predetermined framework. Two researchers independently coded the same subset of transcripts to ensure credibility and reliability. Manual coding was performed line-by-line, assigning descriptive and interpretive codes to segments of text. NVivo 14 software was used to facilitate data organisation and retrieval, while the coding approach remained inductive. The initial coding was conducted independently by two researchers, followed by collaborative meetings to compare interpretations and merge codebooks where appropriate. Inter-coder discussions were documented in analytic memos to provide an audit trail. Discrepancies in coding were discussed until consensus was reached, after which the agreed coding structure was applied to the remaining transcripts. Although coding was primarily inductive, the final thematic map was shaped by both data-driven insights and sensitising concepts from autism diagnosis and health technology adoption literature, reflecting a blended but reflexive analytic stance.

The coding process followed a structured tree: initial open codes were generated inductively from transcripts, which were then grouped into broader categories reflecting barriers, facilitators, and contextual factors. These categories were refined into five overarching themes with subthemes (e.g., within ‘Skepticism and Trust Gaps,’ codes included ‘diagnostic accuracy,’ ‘algorithm opacity,’ and ‘overreliance on AI’). This hierarchical structure ensured transparency and traceability from raw data to final themes.

**Phase 3: Searching for Themes**

Codes were systematically collated into potential themes by grouping related codes that reflected similar meanings or experiences. The team constructed preliminary thematic maps to visualise connections between codes and to identify candidate themes and subthemes. This stage involved clustering codes into broader conceptual categories, which were then labelled as candidate themes. Some codes were assigned to multiple candidate themes initially, allowing for flexibility during subsequent refinement. Cross-case comparisons were undertaken to ensure that emerging themes captured the diversity of perspectives across participant groups. Data collection continued until thematic saturation was reached, meaning no substantially new codes or perspectives were identified in the later focus groups or interviews.

**Phase 4: Reviewing Themes**

Themes were reviewed iteratively against both the coded extracts and the complete dataset to ensure internal coherence and consistency. This process involved refining theme boundaries, combining overlapping categories, and removing any themes that lacked sufficient supporting data. The review process was conducted in two stages: first, at the level of individual coded extracts to ensure they fit meaningfully within each theme; and second, at the level of the overall dataset to verify that themes reflected the broader narratives. Preliminary themes were refined in consultation with a senior qualitative researcher who was not involved in initial coding, providing an external perspective to challenge assumptions and strengthen validity. Peer debriefing sessions with a senior qualitative researcher helped validate the thematic structure.

**Phase 5: Defining and Naming Themes**

Themes were clearly defined to encapsulate their core organising concepts. Theme names were chosen to be concise yet descriptive, retaining the authenticity of participants’ voices while distinguishing between direct accounts and the researchers’ interpretative analysis. Operational definitions were drafted for each theme to delineate their scope and boundaries, specifying inclusion and exclusion criteria for data extracts. To maintain analytic clarity, participant accounts were presented in the results as direct evidence, followed immediately by researcher interpretation in a separate narrative sentence. Any practice or policy recommendations arising from the findings were presented separately in the discussion to avoid conflating interpretation with recommendations in the results section.

**Phase 6: Producing the Report**

The final thematic report integrates both the proportion of participants spontaneously mentioning each issue and rich, illustrative quotes embedded in the analytic narrative. All themes open with a summary paragraph outlining the scope of the theme and the relevant percentages for consistency. Participant quotes are indented and attributed by role and location, immediately followed by interpretive commentary linking the evidence to the analytic points. This ensured that each quote functioned as an integral piece of analytic evidence rather than an isolated anecdote. Consistency was maintained by applying this structure across all themes, enabling comparability and coherence throughout the results section. This approach ensures quotations serve as integrated evidence rather than stand-alone illustrations.

**Trustworthiness Measures**

**Credibility (Internal Validity):** was established through triangulation across FGDs and individual interviews, ensuring that emerging themes were supported by multiple data sources. Member checking was conducted by sharing preliminary interpretations with selected participants, who confirmed accuracy and clarified meanings. Peer debriefing among the research team further strengthened the authenticity of the findings by challenging assumptions and refining interpretations.

**Transferability (External Validity):** was supported by providing thick, contextual descriptions of participant experiences, healthcare barriers, and cultural perceptions of AI-based ASD screening. The inclusion of diverse geographic settings; urban, semi-urban, and rural enabled the identification of context-specific challenges, allowing readers to assess the applicability of findings to similar low-resource settings.

**Dependability (Reliability):** was ensured through detailed documentation of research procedures, including participant recruitment, data collection, and analytic decisions. Independent coding by multiple researchers, followed by consensus meetings, enhanced consistency in theme development. This audit trail allows replication of the analytic process while respecting the reflexive nature of qualitative inquiry.

**Confirmability (Objectivity and Reflexivity):** was strengthened through maintaining a reflexivity journal, where researchers documented assumptions, potential biases, and reflections throughout the study. This practice helped sustain a participant-centered interpretation of the data. Direct participant quotes were integrated into the findings to anchor interpretations in participants’ voices rather than researcher preconceptions.
