# Supplementary material for: Feasibility and Acceptability of AI-Powered Tools for Early Autism Screening in Egypt: Semistructured Focus Group Study
Source: J Med Internet Res. 2026 Apr 7;28:e82564. doi: 10.2196/82564 (PMC13148128; doi:10.2196/82564)
Supplement: Multimedia Appendix 2 [file jmir_v28i1e82564_app2.docx]

**Multimedia Appendix 2**

**Focus Group and Interview Discussion Guide**

This guide was used to structure semi-structured focus group discussions (FGDs) and individual interviews with healthcare professionals and parents of children with ASD. Questions were designed to balance structure with flexibility, allowing participants to elaborate on emerging themes.

**Section 1: Introduction and Rapport Building**

- Brief introduction of moderators and study objectives.
- Assurance of confidentiality and voluntary participation.
- Icebreaker question:
  - *“Can you share a little about your experiences with autism screening or diagnosis, either as a parent or healthcare professional?”*

**Section 2: Current Challenges in Autism Screening**

**Core questions:**

1. *“What challenges do you see in diagnosing autism spectrum disorder (ASD) in Egypt?”*
2. *“What delays or obstacles have you experienced when seeking or providing screening and diagnostic services?”*
3. *“Are there differences in access to autism-related services between urban and rural areas?”*

**Probing prompts (if needed):**

- Long waiting times.
- Shortage of trained specialists.
- Costs of services.
- Travel distance and time burden.
- Diagnostic inconsistency.

**Section 3: Awareness, Stigma, and Community Perceptions**

**Core questions:**

1. *“How is autism understood or perceived in your community?”*
2. *“Have stigma or misconceptions influenced your experience of seeking or providing care?”*

**Probing prompts:**

- Misconceptions about parenting.
- Community-level denial or stigma.
- Perceptions among healthcare professionals (e.g., GPs misinterpreting symptoms).

**Section 4: Perspectives on AI-Powered Screening Tools**

**Core questions:**

1. *“Have you heard of artificial intelligence (AI) tools being used in healthcare? What do you think about applying them to autism screening?”*
2. *“What potential benefits or opportunities do you see in using AI-powered tools for autism diagnosis?”*
3. *“What concerns or risks come to mind?”*

**Probing prompts:**

- Early detection.
- Accessibility for underserved areas.
- Accuracy and reliability.
- Fear of replacing human judgment.
- Data privacy and consent.

**Section 5: Adoption and Implementation Needs**

**Core questions:**

1. *“What conditions would make you feel comfortable using or recommending an AI screening tool?”*
2. *“What features or supports would make AI tools more useful for parents and healthcare workers?”*
3. *“What role should the government, schools, or advocacy groups play in introducing such tools?”*

**Probing prompts:**

- Digital literacy and training.
- Cultural adaptation (language, norms).
- Integration with existing healthcare systems.
- Human oversight and hybrid AI–clinician models.

**Section 6: Wrap-Up**

- Summary of main points discussed.
- Opportunity for participants to add anything they feel was missed:
  - *“Is there anything important we haven’t asked about AI and autism screening that you think should be considered?”*
- Expression of gratitude for participation.
