# Supplementary material for: Feasibility and Acceptability of AI-Powered Tools for Early Autism Screening in Egypt: Semistructured Focus Group Study
Source: J Med Internet Res. 2026 Apr 7;28:e82564. doi: 10.2196/82564 (PMC13148128; doi:10.2196/82564)
Supplement: Multimedia Appendix 3 [file jmir_v28i1e82564_app3.pdf]

Table S3. COREQ (COnsolidated criteria for REporting Qualitative research) checklist

Domain / Topic	Item no.	Guide questions / description	Reported on section
**Domain 1: Research team and reflexivity**			
**Personal characteristics**			
Interviewer/facilitator	1	Which author/s conducted the FGDs and interviews?	Methods – Data Collection; Moderator Training and Power Imbalance Considerations
Credentials	2	What were the researcher's credentials? (e.g., PhD, MD)	Title page; Author affiliations; Authors' contributions
Occupation	3	What was their occupation at the time of the study?	Title page; Author affiliations
Gender	4	Was the researcher male or female?	Methods – Data Collection; Moderator Training and Power Imbalance Considerations
Experience and training	5	What experience or training did the researcher have?	Methods – Data Collection; Moderator Training and Power Imbalance Considerations; Ensuring Trustworthiness
**Relationship with participants**			
Relationship established	6	Was a relationship established prior to study commencement?	Methods – Data Collection; Moderator Training and Power Imbalance Considerations
Participant knowledge of the interviewer	7	What did participants know about the researcher (e.g., personal goals, reasons for doing the research)?	Methods – Recruitment Process; Ethical Considerations
Interviewer	8	What characteristics were reported about	Methods – Moderator Training and Power Imbalance Considerations; Ensuring

Domain / Topic	Item no.	Guide questions / description	Reported on section
characteristics		the interviewer/facilitator (e.g., biases, assumptions, reasons and interests in the research topic)?	Trustworthiness; Discussion – reflexivity paragraph
**Domain 2: Study design**			
**Theoretical framework**			
Methodological orientation and theory	9	What methodological orientation was stated to underpin the study? (e.g., grounded theory, discourse analysis, ethnography, phenomenology, content analysis)	Methods – Data Analysis (reflexive thematic analysis); Introduction & Discussion – use of HBM and TAM
**Participant selection**			
Sampling	10	How were participants selected? (e.g., purposive, convenience, consecutive, snowball)	Methods – Research Design; Inclusion and Exclusion Criteria
Method of approach	11	How were participants approached? (e.g., face-to-face, telephone, mail, email)	Methods – Recruitment Process
Sample size	12	How many participants were in the study?	Abstract; Methods – Research Design; Results – opening paragraph & Table 1
Non-participation	13	How many people refused to participate or dropped out? Reasons?	Methods – Recruitment Process (all invited agreed to participate); Discussion – Limitations (selection bias)

Domain / Topic	Item no.	Guide questions / description	Reported on section
**Setting**			
Setting of data collection	14	Where was the data collected? (e.g., home, clinic, workplace)	Methods – Geographic Distribution & Representation; Data Collection; Table 1
Presence of non-participants	15	Was anyone else present besides the participants and researchers?	Methods – Data Collection (children and, often, the other parent present during parent FGDs)
Description of sample	16	What are the important characteristics of the sample? (e.g., demographic data, clinical data, date)	Methods – Research Design; Inclusion and Exclusion Criteria; Results – first paragraphs; Table 1
**Data collection**			
Interview guide	17	Were questions, prompts, guides provided by the authors? Was it pilot tested?	Methods – Discussion Guide Topics; Multimedia Appendix 2
Repeat interviews	18	Were repeat interviews carried out? If yes, how many?	Methods – Data Collection (no repeat interviews; single FGD per participant; brief follow-up interviews with same participants only)
Audio/visual recording	19	Did the research use audio or visual recording to collect the data?	Methods – Data Recording and Transcription
Field notes	20	Were field notes made during and/or after the interview or focus group?	Methods – Data Recording and Transcription
Duration	21	What was the duration of the interviews or	Methods – Data Collection (60–90

Domain / Topic	Item no.	Guide questions / description	Reported on section
		FGDs?	minutes)
Data saturation	22	Was data saturation discussed?	Methods – Research Design (theoretical saturation); Data Analysis
Transcripts returned	23	Were transcripts returned to participants for comment and/or correction?	Methods – Data Recording and Transcription; Ensuring Trustworthiness (no full transcript return; staged member checking via summaries)
**Domain 3: Analysis and findings**			
**Data analysis**			
Number of data coders	24	How many data coders coded the data?	Methods – Data Analysis (two primary coders + senior qualitative methodologist)
Description of the coding tree	25	Did authors provide a description of the coding tree?	Methods – Data Analysis (example of code → subtheme → theme); Multimedia Appendix 1
Derivation of themes	26	Were themes identified in advance or derived from the data?	Methods – Data Analysis (inductive coding with deductive HBM/TAM overlay); Introduction – theory paragraph
Software	27	What software, if applicable, was used to manage the data?	Methods – Data Analysis (NVivo 14)
Participant checking	28	Did participants provide feedback on the findings?	Methods – Ensuring Trustworthiness (two-stage member checking with subset of parents and clinicians)
Reporting			
Quotations presented	29	Were participant quotations presented to illustrate the themes/findings? Was each quotation identified (e.g.,	Results – Themes 1–5; Methods – Data Recording and Transcription (anonymised identifiers such as "Parent-F, Fayoum"; "Paediatrician-M, Cairo")

**Domain / Topic**	**Item no.**	**Guide questions / description**	**Reported on section**
		participant number)?	
Data and findings consistent	30	Was there consistency between the data presented and the findings?	Results – narrative + Tables 2–6; Discussion – linking themes back to data
Clarity of major themes	31	Were major themes clearly presented in the findings?	Results – headings for Themes 1–5; Tables 2–6; Discussion – opening synthesis of themes
Clarity of minor themes	32	Is there a description of diverse cases or minor themes?	Results – closing paragraph on divergent perspectives; Discussion – sections on AI scepticism vs parental trust, ethical concerns, and urban–rural contrasts
